# Refining a Multicomponent Intervention to Increase Perceived HIV Risk and PrEP Initiation: Focus Group Study Among Black Sexual Minority Men

**DOI:** 10.2196/34181

**Published:** 2022-08-10

**Authors:** Derek T Dangerfield II, Janeane N Anderson, Charleen Wylie, Renata Arrington-Sanders, Ricky N Bluthenthal, Christopher Beyrer, Jason E Farley

**Affiliations:** 1 Johns Hopkins School of Nursing Baltimore, MD United States; 2 Us Helping Us, People Into Living, Inc Washington, DC United States; 3 University of Tennessee Health Science Center Memphis, TN United States; 4 Johns Hopkins School of Medicine Baltimore, MD United States; 5 Keck School of Medicine of the University of Southern California Los Angeles, CA United States; 6 Johns Hopkins Bloomberg School of Public Health Baltimore, MD United States

**Keywords:** sexual health, life course theory, health belief, possible, HIV, preexposure prophylaxis, mHealth, smartphone, health app, digital health

## Abstract

**Background:**

Increased preexposure prophylaxis (PrEP) initiation is needed to substantially decrease HIV incidence among Black sexual minority men (BSMM). However, BSMM perceive others as PrEP candidates instead of themselves and are less likely than other groups to use PrEP if prescribed. Peers and smartphone apps are popular HIV prevention intervention tools typically used independently. However, they could be useful together in a multicomponent strategy to improve perceived HIV risk and PrEP initiation for this group. Information regarding attitudes and preferences toward this multicomponent strategy is limited.

**Objective:**

The goal of this study is to obtain attitudes and perspectives regarding the design of a multicomponent intervention that uses a smartphone app and a peer change agent (PCA) to increase perceived HIV risk and PrEP initiation. The intervention will be refined based on thematic findings for a culturally responsive approach.

**Methods:**

Data were obtained guided by life course theory and the health belief model using 12 focus groups and 1 in-depth interview among HIV-negative BSMM from Baltimore, MD, between October 2019 and May 2020 (n=39). Groups were stratified by the following ages: 18 to 24 years, 25 to 34 years, and 35 years and older. Participants were provided details regarding an existing mobile app diary to self-monitor sexual behaviors and a hypothetical PCA with whom to review the app. Facilitators posed questions regarding perceived HIV risk, attitudes toward the app, working with a PCA, and preferences for PCA characteristics and approaches.

**Results:**

Most participants identified as homosexual, gay, or same gender-loving (26/38, 68%), were employed (26/38, 69%), single (25/38, 66%), and interested in self-monitoring sexual behaviors (28/38, 68%). However, themes suggested that participants had low perceived HIV risk, that self-monitoring sexual behaviors using a mobile app diary was feasible but could trigger internalized stigma, and that an acceptable PCA should be a possible self for BSMM to aspire to but they still wanted clinicians to “do their job.”

**Conclusions:**

HIV-negative BSMM have dissonant attitudes regarding perceived HIV risk and the utility of a mobile app and PCA to increase perceived HIV risk and PrEP initiation. Future research will explore the feasibility, acceptability, and preliminary impact of implementing the multicomponent intervention on perceived HIV risk and PrEP initiation among BSMM in a pilot study.

## Introduction

The United States will not reach its plan to end the HIV epidemic unless HIV incidence among Black sexual minority men (BSMM) substantially decreases [1,2]. Between 2015 and 2019, HIV incidence increased by 6% among BSMM aged 25 to 34 years; in 2019, BSMM accounted for 26% of HIV infections among US gay and bisexual men [1,3]. Approximately 75% of BSMM newly diagnosed with HIV are under the age of 35 years [1]. Preexposure prophylaxis (PrEP) use, however, remains substantially lower for BSMM than for other racial/ethnic groups of gay and bisexual men [4,5]. Increased PrEP initiation is urgently needed to substantially decrease HIV incidence for this group [2,6]. To achieve the US plan to end the HIV epidemic, incidence among BSMM must substantially decrease [1,2]. Between 2015 and 2019, HIV incidence increased by 6% among BSMM aged 25 to 34 years; in 2019, BSMM accounted for 26% of HIV infections among US gay and bisexual men [1,3]. Approximately 75% of BSMM newly diagnosed with HIV are under the age of 35 years [1]. Increased PrEP initiation is urgently needed to substantially decrease HIV incidence for this group [6].

Despite increased awareness, multilevel factors such as insufficient insurance coverage, medication costs, stigma, problems with patient-clinician communication, medical mistrust, concerns regarding side effects, and low perceived HIV risk are barriers to PrEP initiation for BSMM [7-11]. Some BSMM perceive others as at higher risk for HIV infection than themselves and do not view themselves as PrEP candidates because they view their current behaviors as relatively lower risk than their past or their peers [12,13]. Perceived HIV risk among BSMM is also influenced by decreased stigma regarding living with HIV due to data showing that individuals living with HIV can live healthy lives and that individuals with an undetectable viral load cannot transmit the virus [14,15]. However, in a high-prevalence subpopulation, low perceived HIV risk inadequately identifies their objective risk. BSMM with higher perceived HIV risk have greater PrEP interest and use than those with lower perceived HIV risk [16,17]. Since multilevel factors affect sexual behaviors, perceived HIV risk, and PrEP initiation among BSMM, interventions that address these barriers are needed.

Peers and smartphone apps are popular HIV prevention tools. Some interventions leverage in-group members as peer change agents (PCAs) who disseminate health-related information within the population for behavior change [18-21]. Other interventions use apps as electronic diaries because self-monitoring risk behaviors facilitates reflection and reactivity to reduce risks [22-26]. PCAs and apps as intervention tools can address multilevel barriers to PrEP by reducing stigma, circumventing discriminatory health care services, and improving access to care. However, these tools have typically been used independently. Few studies have incorporated PCAs and mobile app–based diaries in a multicomponent strategy, which could be useful to increase perceived HIV risk and PrEP initiation among BSMM.

**Figure 1:**
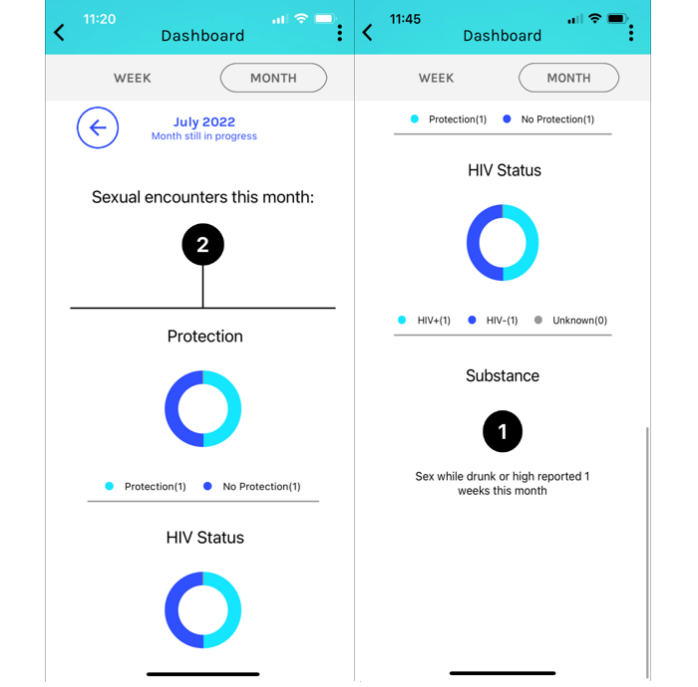
PrEPme smartphone app dashboard.

We designed a multicomponent intervention that consists of an existing smartphone app called PrEPme [18] and a PCA to increase perceived HIV risk and willingness to initiate PrEP among BSMM. Briefly, PrEPme is a smartphone app designed for Maryland users to obtain statewide PrEP service information and navigation support from a community health worker [18]. PrEPme also allows users to self-monitor sexual risk behaviors, view a graph of sexual risk behaviors by week and month (Figure 1), and chat with the in-app community health worker to obtain PrEP service information [18]. In our intervention, we planned for a PCA to meet with HIV-negative BSMM during 2 sessions one month apart. In the baseline session, the PCA would conduct a motivational interview–consistent conversation with HIV-negative BSMM to assess their lifestyles, personal goals and values, HIV risk behaviors, perceived HIV risk, and relative PrEP interest [14,27]. At the end of the session, BSMM would be asked to download PrEPme to record their sexual risk behaviors during the month. In the second session, the PCA would review the diary with BSMM and conduct another motivational interview–consistent conversation to explore motivations for relative HIV risk behaviors, check alignment with goals and values, and assess their PrEP interest. A key utility of the PCA includes sharing health-related information regarding relative and acute risks for HIV, answering questions regarding PrEP efficacy and side effects, and tailoring prevention messaging to improve perceived HIV risk given the information in the PrEPme dashboard. At the end of each session, the PCA would collaborate with BSMM to help interested participants obtain PrEP services.

Guided by life course theory (LCT) [28-30] and the health belief model (HBM) [31,32], we hypothesize that this approach could prove feasible and acceptable, mitigate multilevel barriers, and improve perceived HIV risk and PrEP initiation among BSMM. LCT suggests that age-related differences in exposures to risk, timing of major life events, and accumulated risks impact health behaviors and outcomes [28-30,33]. The HBM posits that perceived disease susceptibility can lead to increased engagement in healthy behaviors [32,34]. Together, this framework informed the design of our multicomponent intervention. Perceived HIV risk and PrEP initiation could be improved by providing BSMM with PrEPme to self-monitor sexual risk and a PCA to change attitudes toward HIV acquisition and introduce PrEP. However, the success of an intervention among BSMM relies upon a culturally responsive strategy prior to its implementation and requires clarity regarding the components and potential barriers [14,35-37]. Therefore, the aim of this study is to explore attitudes toward the intervention guided by our theoretical framework and identify ways to refine the strategy based on feedback from the target population. Findings from this formative research will be used to finalize the study protocol and implement the multicomponent intervention.

## Methods

### Ethics Approval

All study procedures were approved by Johns Hopkins School of Medicine institutional review board (IRB00211578).

### Recruitment and Study Sample

Data were obtained from 12 focus groups and 1 in-depth interview among BSMM from Baltimore, MD, between October 2019 and May 2020 [38]. Participants were recruited using a combination of active (eg, contacting participants from previous studies who provided written consent to be called for future research) and passive (eg, advertising on Craigslist, obtaining participant referrals) strategies [35,37]. Participants were eligible based upon the following criteria: self-identifying as Black or African American, self-identifying as a man, aged 18 years or older, self-reporting as HIV negative, having oral/anal sex with at least 1 male partner in the previous 6 months, and residing in Baltimore, MD.

### Study Procedures

Most focus groups (n=9) were conducted in a private designated research space at Johns Hopkins School of Nursing. The protocol for the last 3 focus groups was updated to a virtual, synchronous format for safety due to COVID-19. Details regarding the protocol for virtual, synchronous focus group conduct have been published elsewhere [35]. The focus group guide was designed using LCT and HBM constructs, and groups were stratified by 3 age groups: 18 to 24 years, 25 to 34 years, and 35 years and older. Groups were limited to 5 or fewer for feasibility and to allow all participants to respond to each question [35,37]. An in-depth interview was conducted because only 1 participant attended a scheduled focus group and still wanted be a part of the study and share thoughts regarding the intervention [14,38]. Two facilitators led the focus groups (and interview). One moderated discussions and recorded notes; the other recorded notes, observed group dynamics, and conducted administrative activities [35,37]. In-person participants provided written informed consent; virtual participants provided oral informed consent that was documented by the study team prior to beginning the focus group [35].

Data collection included reflexive debriefing among the facilitators before and after every focus group to review research intentions, identify potential biases, explore personal challenges of the investigative team, and explore preliminary themes [35,39,40]. Data collection began with a written or online survey via Qualtrics assessing demographic and behavioral characteristics along with perceived HIV risk, assessed using the 8-item Perceived Risk of HIV scale [41], the scores for which ranged from 10 to 40, indicating low-to-high perceived HIV risk. Focus groups were conducted after the survey and lasted 50 to 75 minutes. Facilitators began by asking participants for details regarding their current lifestyles and social activities and how their lives and self-image would change if they received a positive HIV test result to learn more about their perceived severity of disease. Facilitators also targeted domains regarding participants’ thoughts about their behaviors and their risk relative to learning the high HIV prevalence within the city and likelihood of infection for BSMM nationally [6]. Facilitators then shared details regarding PrEPme using handouts and live demonstrations on a smartphone and asked questions regarding attitudes toward using the app to self-monitor sexual risk behaviors. After obtaining attitudes toward the app, facilitators asked questions regarding the usefulness of a PCA in the intervention, attitudes toward sharing their PrEPme diary with the PCA, ideal PCA characteristics, and potential barriers to working with a PCA. All participants were compensated $80 [35].

### Data Analysis

All focus groups were audiorecorded and transcribed verbatim by an IRB-approved company. The first author reviewed all transcriptions for fidelity to audio files and revised text files as needed prior to coding in ATLAS.ti (version 8.4, ATLAS.ti Scientific Software Development GmbH). The facilitators reviewed all focus group recordings, transcripts, and notes and then designed a codebook for descriptive thematic analysis using the questions in the focus group guide (Multimedia Appendix 1). The first author coded transcripts and systematically identified themes using an adapted pile-sorting approach whereby quotes associated with specific codes in ATLAS.ti were into an Excel (Microsoft Corp) spreadsheet and organized [15,40,42]. The facilitators then reviewed the quotes associated with codes and sorted them into piles for similarity within Excel to represent themes. Facilitators identified themes as patterns or novel responses associated with specific codes or focus group questions. To identify a range of themes, novel responses that at least 1 person in the group mentioned were also considered [40,43]. Between-group analysis was conducted to identify potential thematic differences by age.

## Results

### Demographic Characteristics and Descriptive Data

Data resulted in 5 groups of BSMM aged 18 to 24 years, 4 groups aged 25 to 34 years, 3 groups aged 35 years and older, and 1 in-depth interview with a participant aged 18 to 24 years (n=39). All except 1 participant completed the survey (Table 1). Most who completed the survey identified as homosexual, gay, or same gender-loving (26/38, 68%), were employed (26/38, 69%), and single (25/38, 66%). A total of 34% (13/39) of participants had ever been diagnosed with an STI, of whom 38% (5/13) had repeated at least one STI. Perceived Risk of HIV scores ranged from 13 to 40 (x̄=22.4, s=5.3), and HIV Incidence Risk Index for Men Who Have Sex With Men scores ranged from 5 to 28 (x̄=16.2, s=5.7). Most (26/38, 68%) were interested in using a smartphone app to self-monitor their sexual behaviors. Half (19/39, 50%) of surveyed participants had ever used PrEP; 12 out of the 39 reported being current PrEP users.

Focus groups yielded important themes regarding perceived HIV risk and attitudes toward the proposed multicomponent intervention.

**Table 1:** Demographic and behavioral characteristics among Black sexual minority men (n=38).

Variable		Value
**Sexual orientation, n (%)**		
	Heterosexual or straight	2 (5)
	Homosexual, gay, or same gender-loving	26 (68)
	Bisexual	5 (13)
	Pansexual	2 (5)
	Not sure or questioning	1 (3)
**Highest level of education completed, n (%)**		
	Grade 11 or less	2 (5)
	Grade 12 or GED equivalent	16 (42)
	Some college	6 (16)
	Associate degree	3 (8)
	Bachelor’s degree	5 (13)
	Some graduate work	2 (5)
	Graduate degree	4 (10)
**Employment status, n (%)**		
	Full-time	22 (58)
	Part-time	4 (10)
	Unemployed	9 (24)
	Other	3 (8)
**Relationship status, n (%)**		
	Single	25 (66)
	In a committed relationship	8 (21)
	In an open relationship	4 (10)
	Married to a man	1 (3)
**Ever diagnosed with STI^a^, n (%)**		13 (34)
	Repeated STIs	5 (38)
**Last HIV test^b^, n (%)**		
	Never	1 (3)
	Less than 1 month ago	17 (45)
	1-3 months ago	12 (32)
	3 months ago or longer	6 (16)
**App interest, n (%)**		
	Yes	26 (68)
	No	6 (16)
	Don’t know	6 (16)
**Ever used PrEP^c^ (yes), n (%)**		19 (50)
	Current PrEP use (among ever users)	12 (63)
**PrEP telehealth interest, n (%)**		
	Yes	16 (42)
	No	15 (39)
	Don’t know	7 (18)
**PrEP injectable interest, n (%)**		
	Yes	22 (58)
	No	13 (34)
	Don’t know	3 (8)
**Interest in doxycycline for syphilis PrEP, n (%)**		
	Yes	20 (53)
	No	10 (26)
	Don’t know	8 (21)
HIRI-MSM^d^ score, mean (SD)		16.2 (5.8)
Perceived risk of HIV score, mean (SD)		22.4 (5.3)

^a^STI: sexually transmitted disease.

^b^One participant who completed the survey did not provide information regarding their last HIV test.

^c^PrEP: preexposure prophylaxis.

^d^HIRI-MSM: HIV Incidence Risk Index for Men Who Have Sex With Men.

### Low HIV Risk Perceptions While Perceiving Others as Risky

Across and within age groups, participants provided polarizing responses regarding perceived HIV risk. They mentioned that HIV infection would impact their mental health due to lifestyle changes and anticipated stigma from society and potential partners, but they expected to overcome those challenges since contemporary medications improved longevity and quality of life. Despite being presented with Centers for Disease Control and Prevention data regarding HIV prevalence and incidence among BSMM locally and nationally, participants across age groups still perceived their HIV risk as low—even if they disclosed drug use or condomless sex. Each group used positive terms such as goal-oriented and caring to describe themselves and reckless, impulsive, and drug addict to describe individuals they thought were likely to acquire HIV. However, they also mentioned they occasionally had the same characteristics as someone they perceived as high risk for HIV despite not equating their current sexual behaviors as high risk. BSMM in the 2 youngest groups specifically compared their current behaviors to their past and their friends who they believed had riskier behaviors or who had acquired HIV.

### Self-monitoring Sexual Behaviors Triggers Internalized Stigma and Shame

Across groups, participants reported barriers regarding using PrEPme such as remembering to use it and providing honest responses. Those aged 18 to 34 years generally agreed that having a smartphone app diary could be useful for individuals with higher risk behaviors (eg, individuals who use drugs, practice condomless sex with multiple partners), but men age 35 years and older did not think it would be useful or used among BSMM. Participants across all age groups reported that seeing their sexual activities summarized on the dashboard could trigger feelings of internalized stigma regarding their risk behaviors (see Textbox 1).

Participants agreed that having an in-app dashboard of sexual risk behaviors would decrease BSMM’s willingness to provide honest responses and use PrEPme for long. Some added that seeing their condomless sex and receptive anal sex activities in the app could exacerbate internalized stigma and shame that would cause them to discontinue use because society, gay men, and clinicians stigmatize those behaviors.

**Group aged 18 to 24 years (December 14, 2019).**

S2: 42:29 “Some people will have sex to escape from their reality. Some people have sex because they’re sex addicts. Then you see it as offensive because it’s basically in your face like know what you’re doing. You’re constantly reminded that you,—“

S3: 42:42 “You’re being a ‘THOT’”

S2: 42:43 “—you’re being a sexually active person—it’s nothing wrong with having sex. Sex is a natural desire of man. Let’s say I’ve got the app ‘cause I’m trying to work on myself. And I see that I had 12 partners this week. I see that as offensive because now I’m like, ‘All right, I had 12 partners this week,’ and now I start attacking myself... This [gonna] make me feel insecure about myself because it’s right there in front of my face. I can’t deny. I can’t lie to the app.”

### Concerns Regarding Privacy From App Developers and In-App Community Health Workers

The youngest 2 age groups mentioned concerns that their sexual diary could be hacked by app developers or seen by community health workers who were in-app support systems for PrEP linkage. These concerns could be related to anticipated stigma and shame regarding the sexual behaviors documented in the app. However, participants did not mention this explicitly or associate these directly.

### The PCA as a Future Self

Overall, most participants across age groups affirmed that having another BSMM to discuss sexual health and PrEP before visiting a PrEP-prescribing clinician was acceptable if he was relatable and trustworthy. They specified that the PCA should be Black and a current PrEP user so they could share their experiences with side effects and maintaining adherence (see Textbox 2).

These and other characteristics such as aesthetics (eg, style of dress), language use, personal disclosures, and neighborhood familiarity served as indicators for BSMM to use to anticipate how stigmatizing, trustworthy, or relatable the PCA would be.

Despite agreeing that having another BSMM as a PCA was acceptable in a clinical intervention, there were age group differences regarding additional PCA characteristics and the practical role of the PCA. Specifically, those aged 18 to 24 years shared preferences for having a PCA who was older or a Black woman, partly due to anticipated within-group stigma from another BSMM who might judge their sexual behaviors as risky. Older participants shared that they would prefer not to engage with a PCA in practice because they did not want to relieve clinicians of the responsibility of asking detailed and personal questions to know them as patients. They also mentioned concerns that working with a PCA prior to visiting a clinician could be another barrier to PrEP initiation for interested BSMM. Still, the older men agreed that the PCA should be a BSMM who was professional yet relatable to discuss specific cultural issues and PrEP-related concerns such as side effects, efficacy, and relative risks for HIV infection by sexual positioning.

**Group aged 35 years and older (February 1, 2020).**

S3: 35:45 “I feel like some people will share with people that they see some of their self in that person. So, like, for you, for example, I feel more comfortable talking to you about this—”

S6: 35:59 “Yeah.”

S3: 36:00 “—because you are—I do see a lot of myself in you. I know we’re two completely different people but, rather than sitting here talking to like an older straight white lady. It would be a little uncomfortable ‘cause she don’t know this lifestyle like you do. She don’t know what we go through as a young Black gay male.”

S2: 36:24 “Yeah.”

S3: 36:25 “And to even see you have, like, your style—your hair, all that, that helps out... So when you have somebody like you in a room and just to know you a doctor and you still look like me. You’re still young. You groomed, you got the haircut. It just makes you more comfortable.”

S2: 37:19 “Yeah, you right because it took me a long time to open up to my doctor, she’s white.”

S2: 1:01:35 “He made a good example how he sees himself in you—sometimes that’s a good thing as well. You sitting there like, ‘That’s my past and I’m their future self, so I can give a little bit more insight on certain things, so they don’t have to worry about going through what they went through.’”

### Sharing App Data With PCA to Avoid Negative Patient-Clinician Communications

Across groups, participants acknowledged that a benefit of a PCA in this intervention included being able to discuss sexuality freely and trusting the PCA’s cultural competency more than that of a clinician who was not a BSMM (see Textbox 3).

They mentioned that a PCA could help circumvent potentially stigmatizing or unsatisfactory conversations with clinicians regarding sexual risks but were not interested in the PCA discussing health concerns with the clinician on their behalf. Their interest in sharing their PrEPme dashboard with a PCA or clinician was contingent on their perception of how judgmental and trustworthy either would be.

**Group aged 25 to 34 years (May 5, 2020).**

S2: 37:39 “I think that for the sake of like having a real conversation, if it’s somebody that I’m uncomfortable with, I would rather have the conversation in a real tone that’s a peer.”

S3: 37:57 “And someone who looks like me.”

S2: 37:59 “Yeah. And, and they got all the real information, you know what I mean? And they can turn that around and give that to the doctor, because the doctor doesn’t know how to talk to me. The doctor is saying to me, you know, *receptive*, you know what I mean? Versus the peer is saying to me, *bottom*, like, you know. And it’s like, I understand the two roles— like the two roles. But like I don’t think that that’s necessary to repeat myself, if the rapport with the doctor that I have is great. But if the rapport is trash, and that doctor is trash, and I don’t feel comfortable with that doctor, then I’m fine with talking to somebody else to make sure that the point is really internalized.”

## Discussion

### Principal Findings

We designed a multicomponent strategy to increase perceived HIV risk and PrEP initiation among BSMM and obtained in-group perspectives regarding the intervention components for refinement. Overall, BSMM had low-to-moderate perceived HIV risk. Using a PCA as an interventionist could be feasible and acceptable among younger BSMM. However, findings are inconclusive regarding feasibility of using the diary in PrEPme to record and review sexual behaviors for this group. We found age-related differences in attitudes toward the intervention components, which could result from different exposures to HIV-related deaths by age group and changes in personal agency and perceived support needs due to older age [14,44]. Novel insights regarding stigma and shame also emerged that informed considerations for intervention refinement.

BSMM held dissonant attitudes between their sexual behaviors and HIV risk, perceiving their risk as low. Other studies among BSMM have found similar results [17,45]. Having lower perceived HIV risk could be an attempt to combat stereotype threat, which is a perceived risk of conforming to negative stereotypes about a group [46,47] that exacerbates poor patient-clinician communication, refusal of clinical recommendations, and medication nonadherence among Black patients [47,48]. For BSMM, the threat of being stereotypically high risk or sexually deviant could be an important, understudied phenomenon impacting HIV and PrEP initiation. Anticipated and internalized stigma and shame regarding self-monitoring sexual behaviors, discussing sexuality with professionals, and receiving an HIV diagnosis were salient. Additionally, practicing receptive anal sex, or being a bottom, can also carry specific shame and stigma among BSMM due to masculinity stereotypes [49,50]. However, stereotype threat decreases with race-concordant clinicians [43,51]. Therefore, a PCA must be a trained professional with substantial sexual health literacy who understands the issues concerning internalized stigma, shame, and stereotype threat and can help BSMM navigate their perceived HIV risk to make decisions about PrEP. A culturally responsive PCA could circumvent some of the multilevel barriers to perceived HIV risk and PrEP initiation among young BSMM with cultural familiarity, acceptable language, explanation of privacy protocols, and personal disclosures to build trust [35,43].

Findings regarding perceived susceptibility and severity of HIV among BSMM expanded our perspectives regarding the intervention framework. Based upon findings that BSMM had low perceived HIV risk, internalized stigma and shame regarding sexual risk behaviors, and preferences for a culturally congruent PCA, we incorporated possible selves theory [52,53] along with LCT and HBM to refine the intervention strategy. Possible selves represent individuals’ ideas of what they could and want to become in addition to what they are afraid of becoming and can incentivize future behavior [53,54]. Therefore, an intervention using a PCA should consider how congruent with and relatable the peer is with a future self for HIV-negative BSMM beyond race and sexuality concordance [14,35,43]. Factors such as cultural familiarity, professionalism, open-mindedness, sexual health literacy, and PrEP use could be key additional PCA characteristics that build trust, mitigate stereotype threat, and increase willingness to initiate PrEP among BSMM [35,37].

Future research should explore the relative impact of implementing this multicomponent strategy on perceived HIV risk and PrEP initiation among BSMM. Since a sexual health diary app could trigger internalized stigma and clinical research teams are not culturally congruent with BSMM, future research should implement interventions led by culturally congruent investigators familiar with these psychosocial barriers among this population. Future research could explore the potential impact of the dual role of an investigator or clinician as a PCA. Having PrEP clinical trials led by BSMM researchers and clinicians could be an important yet understudied structural, social, and psychological factor in HIV prevention among BSMM.

### Limitations

Limitations should be considered. This study included a convenience sample and is not representative of HIV-negative BSMM. HIV status was self-reported. Groups were intentionally conducted with a small number of participants to increase rapport, trust, and privacy and to ensure that everyone could respond to every question if desired [35,37]. Information regarding implications of the intervention regarding on-demand and injectable PrEP was limited.

### Conclusion

Qualitative studies are crucial to designing culturally relevant interventions for BSMM because they identify how socioecological factors such as stigma impact HIV risk for this group [55]. Despite the proliferation of smartphone apps for health promotion and HIV prevention [56], we found that using PrEPme to record and review sexual risk behaviors may not be as innocuous as anticipated. Therefore, we plan to train the PCA to anticipate stereotype threat and manage insider-outsider dynamics to demonstrate relatability, trustworthiness, care, and professionalism. In the refined intervention, we plan to encourage participants to use the diary in PrEPme and ensure that the PCA is a nonjudgmental possible self who can help BSMM navigate feelings of stigma, shame, low perceived HIV risk, and PrEP hesitancy as participants reflect on their behaviors. Since providing objective HIV risk scores alone does not increase PrEP uptake among BSMM [7], the PCA will be trained in motivational interviewing to partner with BSMM to explore their health goals, perceived HIV risk, and relative interest in PrEP given their responses to the diary in PrEPme. We named the refined intervention strategy *Possible* based upon findings from this study.

## Appendix

Multimedia Appendix 1 Focus group guide.

## Abbreviations

BSMM: Black sexual minority men
HBM: health belief model
LCT: life course theory
NIH: National Institutes of Health
PCA: peer change agent
PrEP: preexposure prophylaxis

## Data Availability

The data set generated and analyzed during this study is not publicly available to protect participant confidentiality but is available from the corresponding author on reasonable request.
