# Supplementary material for: Refining a Multicomponent Intervention to Increase Perceived HIV Risk and PrEP Initiation: Focus Group Study Among Black Sexual Minority Men
Source: JMIR Form Res. 2022 Aug 10;6(8):e34181. doi: 10.2196/34181 (PMC9403828; doi:10.2196/34181)
Supplement: Multimedia Appendix 1 [file formative_v6i8e34181_app1.docx]

Date: ____________ Age Group: ________

1. Introduction of Research Team
2. Overview of goals of study (Learning more about risk perceptions and designing an intervention)
3. Reminder of informed consent
4. Review of Rules (e.g., confidentiality, talking one at a time)
5. Questions from participants
   1. **HIV risk among Black SMM**

*Can I have 3 volunteers to describe your daily routine.*

*Who has a different routine than the ones that have been described?*

*How would your life change if you got an HIV positive test?*

*Probe: Are these changes significant to you? Why or why not?*

**Prompt:** I am going to pass out a sheet of paper. On one side of the paper, I would like you to describe yourself in a few words. On the other side of the paper I would like you to describe some of the characteristics of a person you think is most likely to acquire HIV.

*What characteristics of a person you think is at risk of HIV infection overlap with your personal characteristics?*

*Who thinks their risk for acquiring HIV low?*

*(Probe: Why or why not)*

**Prompt:** There is research that suggests that Black sexual minority men have a 1 in 2 or 50% chance of acquiring HIV in their lifetime. Between 2010 and 2016, HIV infections increased by 40% among Black SMM ages 25 to 34. In Baltimore, more than half of HIV infections are among men who have sex with men.

*Who still thinks their risk for acquiring HIV low?*

*(Probe:* ***Why or why not****)*

- 1. **PrEP Knowledge and Attitudes**

*What do you all know about PrEP?*

**Prompt:** PrEP is used when HIV-negative people take daily medicine to prevent HIV. When taken daily, PrEP is highly effective for preventing HIV. Studies have shown that PrEP reduces the risk of getting HIV from sex by about 99% when taken daily. You **must** take PrEP daily for it to work. PrEP can cause side effects like nausea in some people, but no serious side effects have been observed, even in HIV-negative people who have taken PrEP for up to 5 years.

*What are your concerns about the claims made about PrEP’s effectiveness?*

**Prompt:** Black men like you who have participated in other research studies have said that some of the barriers to taking PrEP are: 1) limited insurance coverage, not wanting to take a daily pill, and not being comfortable talking with healthcare providers about sexuality.

*What are other things that make starting PrEP challenging that we don’t know about?*

*How could a shot that could last up to two months change your willingness to get on PrEP?*

*How can providers talk to you about PrEP in a way that would increase your interest in using it?*

- 1. **Past experiences in healthcare environments**

*What kinds of things do you talk with your provider about?*

*How do characteristics like age, race, gender, or sexual orientation impact your trust in your provider’s practice?*

*How do providers talk to you about your risk for HIV?*

*[ask for a raise of hands of participants who have talked to providers about PrEP]*

*Of those of you who raised your hand, please describe what your experience.*

*How comfortable are you about talking with providers about behaviors like “topping” and “bottoming?” Oral sex?*

*How do discussions of sexual behaviors like topping and bottoming with past providers impact your clinic visits?*

**Prompt:** Please take a moment to look at the questions I have provided. These questions are used to help healthcare providers assess HIV risk for sexual minority men, including gay and bisexual men.

*What do you think about how these questions ask Black sexual minority men about “topping” and “bottoming”?*

*How else could a provider ask patients who are Black sexual minority men about these sexual behaviors?*

*What is missing from these questions that would help providers better learn if Black sexual minority men are engaging in HIV risk?*

*How else could providers ask these questions in a way that would help build trust from Black sexual minority men?*

- 1. **Intervention Design**

**Prompt:** We are interested in seeing if there is a new way to support Black men in assessing HIV risks and promoting sexual health using PrEP.

(Provide brief overview of the intervention that includes a mobile app to help increase HIV risk perceptions.)

*What are your favorite features of the apps that you use the most?*

**Prompt**: The app is password protected and will allow you to record your sexual behaviors and ask you whether you used drugs or alcohol before or during sex. If you do not record anything, there is a notification that will come up to remind you every week. At the end of the month, it will show you a total number of sexual partners and the amount of substance use before or during sex. Like how a calorie app helps people track their calorie intake for the week/month.

Show photos/demonstration of the PrEPme app.

*What do you think about an app that will help you monitor sexual risks?*

*What are concerns about using an app like this?*

*What are potential barriers to using an app like that? How can we overcome them?*

*What do you think about sharing the information in the app with a healthcare provider?*

*(Probe: What if there was an app like this you could use before your visit to provide information to your provider on your behalf?)*

*What do you think about talking with someone within the app who could help you learn more about and possibly obtain PrEP?*

*What do you think about sharing the information in the app with another Black SMM during sexual healthcare visits instead of a provider?*

*Who might this person be relative to you? Someone you know? A friend? Any Black MSM?*

*What kind of information about your sexual behaviors would you be willing to share with this individual?*

*What other characteristics of a provider would impact your willingness to share the information in the app?*

*What other information about HIV should providers talk with you about to help you identify risks?*

*For this kind of study, we will providing incentives for each study visit, which could be $50 gift cards or $50 in cash. Which would be preferred?*

*Probe: Would you consider transportation as an incentive?*

*How would weekly reminders from a research team member about using the app impact your interest in using it?*

**Closing *-*** *Review of comments. Thank participants for time. Reminder of confidentiality*
